# Reducing the nicotine content of tobacco by grafting with eggplant

**DOI:** 10.1186/s12870-020-02459-4

**Published:** 2020-06-22

**Authors:** Mengjuan Ren, Mengyue Zhang, Huijuan Yang, Hongzhi Shi

**Affiliations:** College of Tobacco Science/Tobacco Cultivation Key Laboratory of China Tobacco/Tobacco Harm Reduction Research Center, Henan Agricultural University, No. 95 Wenhua Road, Zhengzhou, 450002 Henan Province China

**Keywords:** Nicotine content, Tobacco, Grafting, Differentially expressed genes, Transcription factors

## Abstract

**Background:**

Nicotine is a stimulant and potent parasympathomimetic alkaloid that accounts for 96–98% of alkaloid content. A reduction in the amount of nicotine in cigarettes to achieve a non-addictive level is necessary. We investigated whether replacing tobacco root with eggplant by grafting can restrict nicotine biosynthesis and produce tobacco leaves with ultra-low nicotine content, and analyzed the gene expression differences induced by eggplant grafting.

**Results:**

The nicotine levels of grafted tobacco leaves decreased dramatically. The contents of nornicotine, anabasine, NNN, NNK, NAT, total TSNAs and the nicotine of mainstream cigarette smoke decreased, and the contents of amino acids and the precursors of alkaloids increased in grafted tobacco. Eggplant grafting resulted in the differential expression of 440 genes. LOC107774053 had higher degrees in two PPI networks, which were regulated by LOC107802531 and LOC107828746 in the TF-target network.

**Conclusions:**

Replacing tobacco root with eggplant by grafting can restrict nicotine biosynthesis and produce tobacco leaves with ultra-low or zero nicotine content. The differential expression of LOC107774053 may be associated with eggplant grafting.

## Background

Alkaloids are a class of naturally occurring organic compounds that primarily contain basic nitrogen atoms [1]. Nicotine is a stimulant and potent parasympathomimetic alkaloid found in the nightshade family of plants, *Solanaceae*, and a stimulant drug present in tobacco [2]. Additionally, nicotine is the most important neuroteratogen component of tobacco smoke [3]. As the main active component in tobacco leaves, nicotine can induce addiction [4, 5]. Benowitz et al. [6] first put forward the idea that reducing tobacco nicotine content below the threshold of addiction could effectively reduce smokers’ dependence on tobacco. The food and drug administration has considered forcing a reduction in the amount of nicotine in cigarettes to achieve a non-addictive level [7]. The World Health Organization suggests that the nicotine content in cigarette tobacco should be reduced below 0.4 mg/g [8] to between 0.2 and 0.3 mg/g, which is lower than the threshold of maintaining addiction [6]. Therefore, studies relating to low/ultra-low nicotine tobacco technology and its influence on tobacco quality are urgently needed.

Current techniques for reducing nicotine content in tobacco include mutation breeding [9], chemical and agronomic regulation [10], and industrial methods [11]. However, these methods have some limitations. For example, the range of chemical and agronomic regulation to reduce nicotine content in tobacco leaves is very limited [12]. Inhibition or knockout of the key genes of nicotine synthesis can effectively reduce nicotine synthesis and content in tobacco leaves to levels of near zero, but it is difficult to apply this in production due to the impact on quality and restrictions on genetically modified organisms [13, 14]. Industrial methods, such as chemical extraction, can reduce nicotine content in tobacco leaves to very low levels, but they also have adverse effects, such as the decrease of aroma components [11].

Grafting can delay the occurrence of tobacco bacterial wilt, reduce the incidence and disease index, and increase the yield, output value, and average price of tobacco plants [15]. The synthetic site of tobacco nicotine is located at the root [16]. The resistance of grafted eggplant seedlings with tobacco as scion to bacterial wilt is increased [17]. In 1942, Dawson [16] grafted tobacco on tomato and found that there was no significant accumulation of nicotine in tobacco leaves or stems when the tobacco scion was grown on tomato rootstock. Because this study mainly focused on the distribution of nicotine between stock and scion in reciprocal grafts of tobacco and tomato, there was no experiment about chemical composition detection as well as gene expression changes.

Therefore, in the current study, using flue-cured tobacco as scion and eggplant of the same family as rootstock, the interspecific grafting technology of tobacco and eggplant was explored, and the agronomic characteristics and chemical composition of fresh and roasted tobacco were determined during the growth and development of the plants. The effects of ultra-low nicotine content on the sensory quality of tobacco leaves and some harmful components in tobacco leaves and smoke were clarified. Furthermore, the gene expression differences induced by eggplant grafting were explored via bioinformatics analyses for the tobacco/tobacco and tobacco/eggplant groups, which included principal component analysis (PCA), differentially expressed genes (DEGs) analysis, gene ontology (GO) and Kyoto Encyclopedia of Genes and Genomes (KEGG) pathway enrichment analysis, protein-protein interaction (PPI) network analysis, and transcription factors (TFs)-target network analysis. We aimed to study whether replacing tobacco root with other species by grafting restricts nicotine biosynthesis and produces tobacco leaves with ultra-low or zero nicotine content.

## Results

### Agronomic traits

**Fig. 1:**
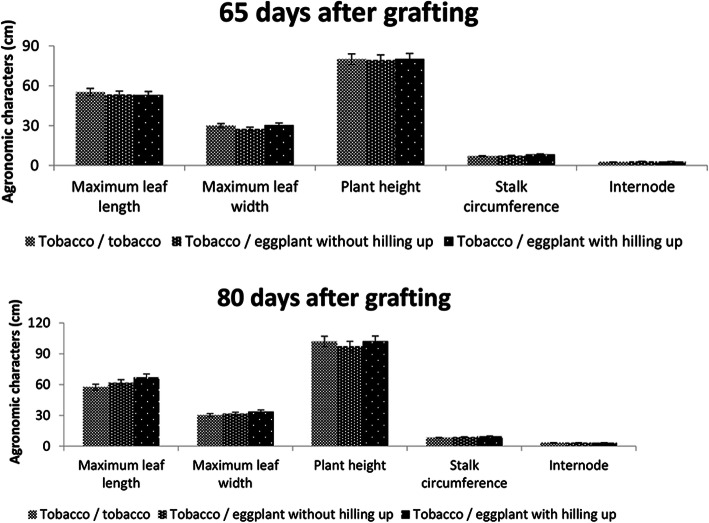
The effect of tobacco and eggplant grafting on agronomic traits of flue-cured tobacco

There were no significant differences in maximum leaf length and width, plant height, stalk circumference, or internode between tobacco/tobacco and tobacco/eggplant groups without hilling up as well as tobacco/eggplant with hilling up 65 and 80 days after grafting (*P* < 0.05) (Fig. 1).

### Alkaloids

The alkaloid contents in tobacco leaves significantly decreased and the proportion of anabasine to total alkaloids increased with tobacco and eggplant grafting. Nornicotine and anatabine contents were not detectable in fresh leaves produced by tobacco and eggplant grafting. The nicotine levels of grafted tobacco leaves decreased dramatically, with nicotine content in fresh tobacco leaves being lowered to 0.08% and in flue-cured leaves to 0.09% (decreased 95% compared to tobacco/tobacco control). The proportion of nicotine to total alkaloids decreased, and the proportions of nornicotine, anabasine, and anatabine in total alkaloids increased in fresh and cured tobacco leaves (Tables 1 and 2).

**Table 1:** Effect of tobacco and eggplant grafting on alkaloid contents in fresh tobacco

Time/d	Treatments	Alkaloid content (%)					Percentage of total alkaloids (%)			
		Nicotine	Nornicotine	Anabasine	Anatabine	Total alkaloids	Nicotine	Nornicotine	Anabasine	Anatabine
65	Tobacco/tobacco	0.4876 ± 0.02aA	0.0239	0.0052 ± 0aA	0.0249	0.5416 ± 0.01aA	90.03	4.41	0.96	4.6
	Tobacco/eggplant without hilling up	0.0834 ± 0cC	–	0.0049 ± 0bB	–	0.0883 ± 0cC	94.45	–	5.55	–
	Tobacco/eggplant with hilling up	0.1416 ± 0bB	–	0.0048 ± 0bB	–	0.1464 ± 0.01bB	96.72	–	3.28	–
80	Tobacco/tobacco	0.6401 ± 0.01aA	0.0283	0.0046 ± 0aA	0.0317	0.7047 ± 0.01aA	90.83	4.02	0.65	4.5
	Tobacco/eggplant without hilling up	0.0804 ± 0cC	–	0.0034 ± 0cC	–	0.0838 ± 0cC	95.94	–	4.06	–
	Tobacco/eggplant with hilling up	0.1507 ± 0bB	–	0.0039 ± 0bB	–	0.1546 ± 0.01bB	97.48	–	2.52	–

**Note:** The difference between treatments is significant (*P* < 0.05) if the same number is not marked with the same lowercase letter and the difference between the treatments is extremely significant (*P* < 0.01) without the same capital letter

**Table 2:** Effect of tobacco and eggplant grafting on alkaloid contents in cured tobacco

Position	Treatments	Alkaloid content (%)					Percentage of total alkaloids (%)			
		Nicotine	Nornicotine	Anabasine	Anatabine	Total alkaloids	Nicotine	Nornicotine	Anabasine	Anatabine
Upper leaf	Tobacco/tobacco	1.9237 ± 0.02aA	0.0460 ± 0aA	0.0175 ± 0aA	0.0672 ± 0aA	2.0544 ± 0.02aA	93.64	2.24	0.85	3.27
	Tobacco/eggplant without hilling up	0.0999 ± 0cC	0.0037 ± 0cC	0.0087 ± 0cC	–	0.1123 ± 0cC	88.96	3.20	7.84	–
	Tobacco/eggplant with hilling up	0.2742b ± 0B	0.0109 ± 0bB	0.0168 ± 0bB	0.0118 ± 0bB	0.3137 ± 0.01bB	87.41	3.47	5.36	3.76
Middle leaf	Tobacco/tobacco	1.9423 ± 0.03aA	0.0664 ± 0aA	0.0110 ± 0bB	0.0714 ± 0aA	2.0911 ± 0.03aA	92.88	3.18	0.53	3.41
	Tobacco/eggplant without hilling up	0.1068 ± 0cC	–	0.0094 ± 0cC	–	0.1162 ± 0cC	91.91	–	8.09	–
	Tobacco/eggplant with hilling up	0.3205 ± 0bB	0.0134 ± 0bB	0.0228 ± 0aA	0.0138 ± 0bB	0.3705 ± 0.01bB	86.5	3.62	6.15	3.73

**Note:** The difference between treatments is significant (*P* < 0.05) if the same number is not marked with the same lowercase letter and the difference between the treatments is extremely significant (*P* < 0.01) without the same capital letter

### Feeding selection behavior

The results of feeding selection behavior showed that (Fig. S1), under the same conditions, the ultralow nicotine tobacco/eggplant leaves were preferred, and the feeding area of the tobacco/eggplant increased significantly over time. However, there was no significant difference in the feeding area of the control. When the leaf area of low nicotine tobacco was zero, the *Heliothis assulta* began to eat the control leaves.

### Main chemical constituents

Significant differences were found for fresh tobacco in total nitrogen, total sugar, reducing sugar, and nicotine after grafting (*P* < 0.05). The total nitrogen contents increased, and total sugar, reducing sugar, and nicotine significantly decreased in tobacco/eggplant with hilling up. After grafting, significant differences were observed in the chemical composition of cured tobacco (*P* < 0.05). Nicotine was 95% lower than the control in both the upper and middle leaves. Starch of the upper and middle leaves increased by 79 and 55% for tobacco/eggplant without hilling up compared to the control, respectively (Tables 3 and 4).

**Table 3:** Effect of tobacco and eggplant grafting on the chemical composition of fresh tobacco

Time /d	Treatments	Total nitrogen	Total sugar	Reducing sugar	Nicotine
65	Tobacco / tobacco	3.50 ± 0.15cB	8.23 ± 0.30aA	7.79 ± 0.10aA	0.75 ± 0.02aA
	Tobacco / eggplant without hilling up	4.17 ± 0.08bA	7.32 ± 0.27bB	6.78 ± 0.33bB	0.07 ± 0cC
	Tobacco / eggplant with hilling up	4.35 ± 0.19aA	7.35 ± 0.33bB	5.93 ± 0.27cC	0.15 ± 0.01bB
80	Tobacco / tobacco	2.54 ± 0.01cC	8.43 ± 0.22aA	6.10 ± 0.17aA	1.16 ± 0.04aA
	Tobacco / eggplant without hilling up	2.84 ± 0.02aA	5.78 ± 0.13cC	3.90 ± 0.10cC	0.06 ± 0cC
	Tobacco / eggplant with hilling up	2.71 ± 0.10bB	6.75 ± 0.24bB	5.39 ± 0.21bB	0.21 ± 0.01bB

**Note:** The difference between treatments is significant (*P* < 0.05) if the same number is not marked with the same lowercase letter and the difference between the treatments is extremely significant (*P* < 0.01) without the same capital letter

**Table 4:** Effect of tobacco and eggplant grafting on the chemical composition of cured tobacco

Position	Treatments	Protein	Total sugar	Reducing sugar	Nicotine	Total nitrogen	Starch
Upper leaf	Tobacco / tobacco	13.89 ± 0.33bA	19.30 ± 0.18aA	17.17 ± 0.25aA	1.89 ± 0.03aA	3.11 ± 0.03cB	3.05 ± 0.08cC
	Tobacco / eggplant without hilling up	14.15 ± 0.07aA	18.12 ± 0.47bB	15.84 ± 0.12cB	0.10 ± 0.01bB	3.21 ± 0.01aA	5.46 ± 0.05aA
	Tobacco / eggplant with hilling up	14.26 ± 0.21aA	18.98 ± 0.30aA	16.13 ± 0.10bB	0.27 ± 0.01bB	3.15 ± 0.07bA	3.41 ± 0.14bB
Middle leaf	Tobacco / tobacco	12.27 ± 0.43bB	16.87 ± 0.44aA	15.79 ± 0.25aA	2.00 ± 0.04aA	2.34 ± 0.03bB	3.41 ± 0.03cB
	Tobacco / eggplant without hilling up	13.60 ± 0.30aA	14.88 ± 0.02bB	12.77 ± 0.18bB	0.11 ± 0.01bB	2.45 ± 0.05aA	5.29 ± 0.22aA
	Tobacco / eggplant with hilling up	13.85 ± 0.12aA	14.00 ± 0.05bB	12.26 ± 0.32bB	0.31 ± 0.02bB	2.49 ± 0.08aA	3.67 ± 0.16bB

**Note:** The difference between treatments is significant (*P* < 0.05) if the same number is not marked with the same lowercase letter and the difference between the treatments is extremely significant (*P* < 0.01) without the same capital letter

After grafting, significant differences were observed in amino acids between tobacco with eggplant grafting and the control in fresh and cured tobacco leaves (*P* < 0.05). In fresh tobacco leaves, except for the contents of Tyr, the amino acid contents of the tobacco leaves grafted with eggplant were significantly higher than the control. In cured tobacco leaves grafted with eggplant, significant differences were observed in amino acids (*P* < 0.05), and the contents of Glu, Tyr, and Phe in tobacco/eggplant without hilling up were the lowest. According to the total amino acid contents, there were significant differences between tobacco/eggplant without hilling up as well as tobacco/eggplant with hilling up and tobacco/tobacco (*P* < 0.05). No significant difference between tobacco/eggplant without hilling up and tobacco/eggplant with hilling up were observed, and the total amino acid contents of tobacco/eggplant treatments were significantly higher than the control (Tables S1 and S2). In summary, tobacco and eggplant grafting was conducive to the accumulation of amino acids.

For the 4-(methylnitrosamino)-1-(3-pyridyl)-1-butanone (NNN), (R,S)-N-nitrosoanatabine (NAT), (R,S)-N-nitrosoanabasine (NAB), 4-(methylnitrosamino)-1-(3-pyridyl)-1-butanone (NNK), and total TSNAs contents of tobacco produced by grafting, there were significant differences compared to the control (*P* < 0.01). The NNN, NNK, and total TSNA contents of the upper leaf in the tobacco/eggplant without hilling up were 82.71, 79.46, and 68.78% lower than for the control, respectively. The NNN, NNK, and total TSNA contents of the middle leaf in the tobacco/eggplant without hilling up were 79.68, 79.80, and 68.63% lower than in the control, respectively. The decrease of total TSNA content was mainly caused by the decrease of NNN and NNK contents (Table S3).

### Characteristics of smoke and changes of sensory response

**Fig. 2:**
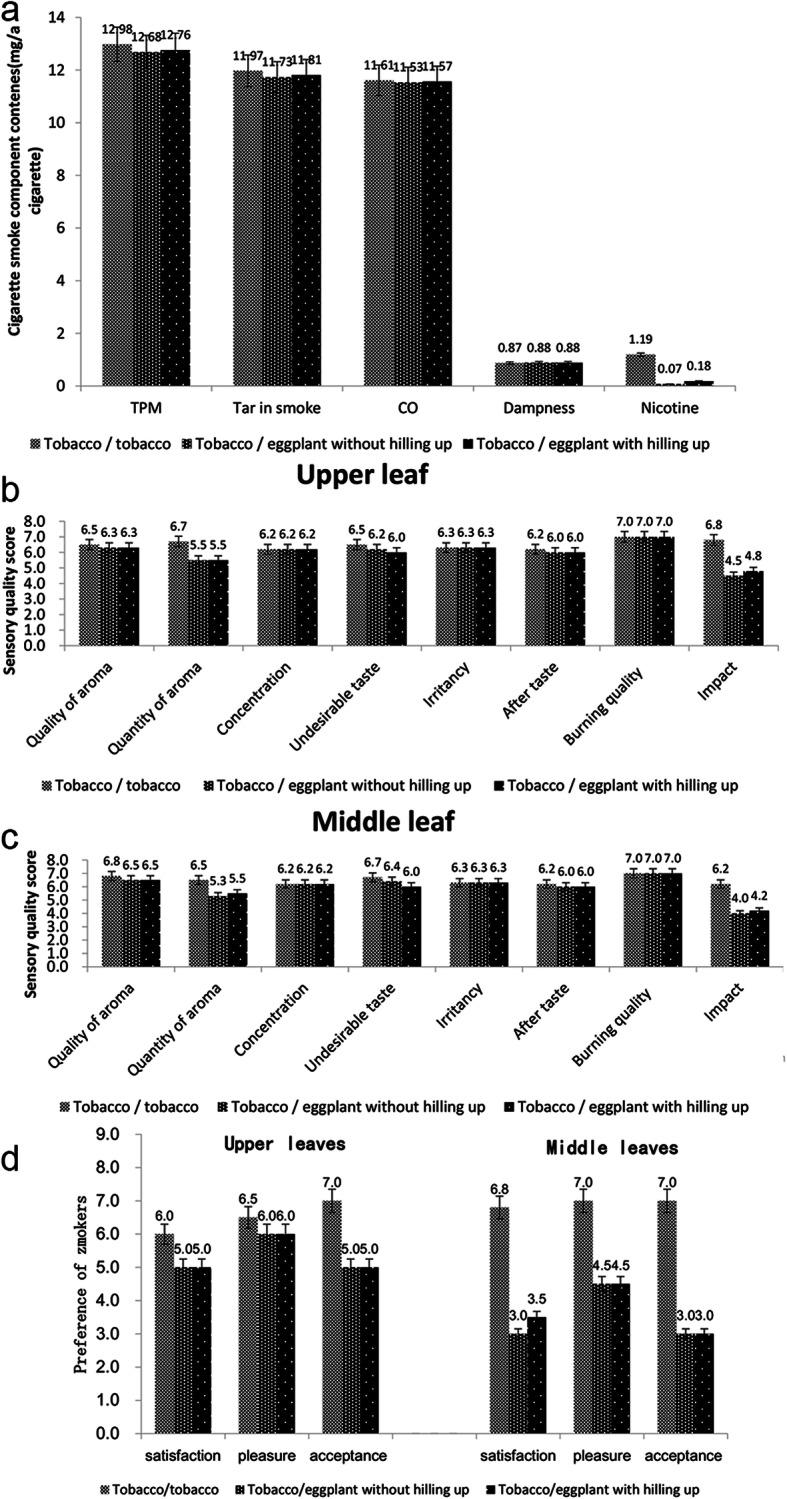
**a**: The effect of tobacco and eggplant grafting on the composition of mainstream cigarette smoke; **b**: The effect of tobacco and eggplant grafting on the sensory quality in upper tobacco leaves; **c**: The effect of tobacco and eggplant grafting on the sensory quality in middle tobacco leaves; **d**: The effect of tobacco and eggplant grafting on the response of smokers

There was no significant difference in the total particulate matter, tar in smoke, or carbon monoxide emissions among tobacco/tobacco, tobacco/eggplant without hilling up, and tobacco/eggplant with hilling up treatments (*P* < 0.05). There were significant differences in nicotine release compared to the control (*P* < 0.01). The nicotine release of the tobacco/eggplant without hilling up and tobacco/eggplant with hilling up were 94.12 and 92.66% lower than tobacco/tobacco, respectively. The results showed that tobacco and eggplant grafting can control nicotine release in cigarette smoke to a large extent (Fig. 2a).

For the effect of tobacco and eggplant grafting on the sensory quality, the upper and middle leaves of cured tobacco were the same. Compared to the control, there was a smaller reduction in aroma quality, undesirable taste, and after taste in the tobacco/eggplant with hilling up and tobacco/eggplant without hilling up, and there were no differences in concentration, irritancy, and burning quality. After grafting with eggplant, there were significant changes in the quality of the aroma and impact (Fig. 2b, c).

The effect of upper leaves on the response of smokers was the same with middle leaves. Compared to the control, the satisfaction, pleasure, and acceptance of the tobacco/eggplant with hilling up and tobacco/eggplant without hilling up were lower than the control. The scores of satisfaction, pleasure, and acceptance between the tobacco/eggplant with hilling up and tobacco/eggplant without hilling up were the same. The results showed that the ultra-low nicotine tobacco produced by the grafting of tobacco and eggplant had an effect on the smokers’ reactions (Fig. 2d).

### Data preprocessing

A total of 49.54 G raw data were obtained through sequencing, and 48.80 G clean data were obtained after screening. After data preprocessing, 45,956 genes were obtained for the present study.

### Correlation analysis and PCA between samples

The correlation coefficient P between the samples was 0.982–1. The mean of similarity in the intra-group sample was 0.9999, and in the inter-group sample was 0.983 (Fig. S2a). PCA results showed that the tobacco/tobacco group was obviously separated from the tobacco/eggplant group (Fig. S2b).

### DEGs analysis

After analysis, 440 DEGs (212 up- and 228 down-regulated) were obtained between the tobacco/tobacco group and tobacco/eggplant group 2. The heatmap and volcano plot (Fig. S3) indicated that DEGs could separate the samples completely according to pre-grouping (tobacco/tobacco group and tobacco/eggplant group), suggesting that these genes were associated with different alkaloid synthesis capabilities.

### GO and KEGG pathway enrichment analyses

The analyses showed that 440 DEGs were enriched in 78 MF, 23 CC, 180 BP, and 12 KEGG pathways. The top 10 GO and all KEGG pathways are presented in Fig. S4. The genes were significantly enriched in the pathway of vitamin B6 metabolism, transporters, and riboflavin metabolism, and the BP of the cellular response to phosphate starvation, cellular response to external stimulus, and phosphate ion homeostasis.

### PPI network construction and network degree analysis

PPI networks for *Nicotiana sylvestris* (Fig. S5a) and *Nicotiana tomentosiformis* (Fig. S5b) were constructed. There were 113 protein nodes and 167 protein pairs in the PPI network for *N. sylvestris*. There were 104 protein nodes and 142 protein pairs in the PPI network for *N. tomentosiformis*. After network degree analysis, the corresponding proteins of LOC107758934, LOC107774053, LOC107792960, LOC107820178, LOC107821210, and LOC107824707 had higher degrees in two networks and might be hub proteins.

### PPI module analysis

A module was identified in PPI for *Nicotiana sylvestris* (Fig. S6a). It included 10 proteins (nine up- and one down-regulated) corresponding to 13 genes. GO and KEGG pathway analyses showed that these 13 genes were enriched in 27 MF, 17 CC, 58 BP (e.g., nucleosome assembly, chromatin assembly, and protein-DNA complex assembly), and two KEGG (chromosome and associated proteins and exosome) pathways. The top 10 results are presented in Fig. S6b.

### TFs prediction and TF-target network construction

TFs were predicted and the TF-target network is presented in Fig. S7. The TF-target network includes seven TFs (LOC107789709, LOC107827800, LOC107802531, LOC107828746, LOC107777554, LOC107767686, and LOC107765920) and 58 differentially expressed target genes regulated by these TFs. LOC107774053, regulated by LOC107802531 and LOC107828746, had higher degrees in the PPI network.

## Discussion

Our present grafting results show that the nicotine levels of grafted tobacco leaves decreased dramatically, while the botanical and agronomic characteristics of the tobacco leaves did not change significantly in the tobacco and eggplant grafting group. The contents of nornicotine and anabasine decreased, and the contents of amino acids and the precursors of alkaloids increased significantly in grafted tobacco. Higher levels of protein, starch, and total nitrogen and lower levels of sugar contents were also observed. The contents of NNN, NNK, NAT, total TSNAs, and the nicotine of mainstream cigarette smoke decreased significantly. Bioinformatics analysis revealed 440 DEGs in the leaves of grafted tobacco, which were significantly enriched in the pathways of vitamin B6 metabolism and transporters and riboflavin metabolism. LOC107774053 had higher degrees in two PPI networks, which were regulated by LOC107802531 and LOC107828746 in the TF-target network.

In this study, the growth appearance and characteristics of grafted tobacco were similar to those of the control. The tobacco grafted with eggplant had an effect on the contents of the chemical components in tobacco leaves, and the contents of the amino acids and protein in grafted tobacco were higher than in the control. The decrease in sugar content may be correlated with the distribution and metabolism changes of carbon and nitrogen. The amino acids of the tobacco grafted with eggplant may not enter the alkaloid synthesis pathway and may be used more in protein synthesis or other metabolic pathways. The low content of nicotine was still detected in the tobacco/eggplant without soil covering, which indicated that it had a weak ability for nicotine synthesis. However, further studies are required regarding whether the trace nicotine came from the eggplant or grafted sections. This result was in accordance with the findings of Dawson [16]. There was no significant accumulation of nicotine in tobacco leaves or stems when the tobacco scion grew on tomato rootstock. He also found that the nicotine remained in the stem and lower leaves, initially present in the scion, and leaves and stem tissues that subsequently developed were nicotine-free. In our study, adventitious roots were induced from the base of scion by culturing, and the nicotine content of the tobacco leaves increased significantly compared to that of the grafted tobacco without soil, indicating that the adventitious roots of scions could synthesize nicotine; thus, the soil culture affected the nicotine synthesis of adventitious roots. In addition, the content of anabasine in grafted tobacco leaves did not decrease synchronously with the content of nicotinic acid, but its proportion in total alkaloid content increased significantly, which might be due to anabasine having a synthetic ability in leaves [18].

For the present study, the contents of NNN, NNK, NAT, and total TSNAs decreased significantly with the decrease in nicotine content after grafting with eggplant. Furthermore, the total particulate matter, tar, and carbon monoxide in cigarette smoke after grafting were similar to those of the control, and the nicotine content in flue gas was significantly lower than in the control. After reducing the nicotine content of tobacco leaves by grafting technology, the aroma quality and quantity, undesirable taste, impact, irritancy, satisfaction, pleasure, and acceptance decreased. Nicotine and alkaloids are the precursors of TSNAs, and there is a direct correlation between tobacco alkaloids and TSNAs [19, 20]. There was a significant positive correlation between the content of TSNAs and alkaloids in tobacco leaves under the same conditions of nitrate nitrogen content and a modulating environment [21]. The contents of nicotine and nornicotine were positively correlated with the contents of NNN and TSNA [22]. Nicotine and total nitrogen contents in tobacco leaves are the main influencing factors of tobacco internal quality and sensory quality [23]. Nicotine was the main chemical component restricting the taste quality of flue-cured tobacco [24]. Based on conventional breeding and chemical extraction, the quality and sensory quality of ultra-low nicotine tobacco leaves is poor, thus, the consumer acceptance degree is reduced, and it is difficult for this to be used in cigarette formulations [25]. The contents of nicotine in flue-cured tobacco were correlated with aroma quality and the neutral aroma substance content of tobacco leaves [23]. Due to the low nicotine content, it cannot meet the physiological requirements for smoking. The satisfaction and pleasure of smokers are not met, which affects the acceptance of the tobacco leaves. Therefore, the decrease of nicotine content has an effect on the quality of TSNA, aroma, and sensory quality of tobacco leaves.

In the present bioinformatics analysis, the PPI module analysis showed that DEGs were significantly enriched in the BPs of nucleosome assembly, chromatin assembly, and protein-DNA complex assembly, and chromosome pathways and associated proteins and exosomes. Nucleosome assembly following DNA replication, DNA repair, and gene transcription is critical for the maintenance of genome stability and epigenetic information [26]. Chromatin assembly is a fundamental biological process that is essential for the replication and maintenance of the eukaryotic genome [27]. As mentioned above, nicotine was synthesized in the tobacco root [16], which may explain the phenomenon that none of the enriched KEGG pathways and GO terms of DEGs were related to nicotine biosynthesis. LOC107774053 had higher degrees in two PPI networks, which were regulated by LOC107802531 and LOC107828746 in the TF-target network. Therefore, we speculated that the differentially expressed LOC107774053 after eggplant grafting may be regulated by LOC107802531 and LOC107828746. Further studies are needed to verify our conjectures.

## Conclusions

In conclusion, replacing tobacco root with eggplant by grafting can restrict nicotine biosynthesis and produce tobacco leaves with ultra-low or zero nicotine content. However, the underlying molecular mechanisms need to be verified.

## Methods

### Study design and materials

A pot experiment was conducted in 2017 using eggplant (*Solanum melongena* L.) as rootstock and flue-cured variety Yunyan 87 as scion. There were three groups: (1) the control group of tobacco/tobacco, both scions and rootstock were Yunyan 87; (2) tobacco (scion)/eggplant (rootstock) group 1 without hilling up and without soil covering the bottom of the scion; (3) tobacco (scion)/eggplant (rootstock) group 2 with hilling up and with soil covering beyond the grafting interface to induce the adventitious roots of tobacco. The plant materials used in this study were obtained from the Luoyang Tobacco Company and Henan Agricultural University.

### Selection behavior of Heliothis assulta

This experiment was carried out in a 39 × 54 cm foam incubator with humidity maintained at a constant level to prevent the tobacco leaves from wilting. The fresh tobacco leaves (top tender leaves) were collected and the treated and control leaves were cut into 10 equal portions (2 × 12 cm), respectively, arranging according to ABABAB … (Fig. S8). The distance between the two adjacent leaves was the same, with an interval of 2 cm. A total of 25 *H. assulta* of the same size were placed into the center of the box, which was sealed and observed every 3 h. After 12 h, the remaining leaf area was measured using transparent coordinate paper (Fig. S8).

### Fresh tobacco samples

After grafting for 65 or 80 days, the middle leaves (the 12th leaf) were taken from each group. When the oven temperature reached 105 °C, the tobacco leaves were placed into the oven for approximately 15 min to kill the leaf, which was then dried at 50 °C. After grinding, 60 mesh sieves were used to determination the chemical composition, alkaloids, amino acids, and tobacco-specific nitrosamines (TSNAs).

### Flue-curing tobacco samples

Mature tobacco leaves were normally harvested. The middle leaves (7–14 leaf position) and upper leaves (15–20 leaf position) of the tobacco leaves were selected for each treatment. After the tobacco leaves were made into the pole, a label was added, and the three-stage flue-curing process was used to cure the leaves. Cured samples were removed, oven-dried at 50 °C, ground, and filtered through 60 mesh sieves.

### Statistical analysis

Statistical analysis was conducted using Excel 2010 and SSP17.0 software. The Duncan method was used for multiple comparisons.

### RNA-seq and data preprocessing

Samples (tobacco/tobacco group and tobacco/eggplant group 2) were collected from the leaves of *N. tabacum* (common tobacco) with different alkaloid synthesis ability. Six samples (tobacco/tobacco 1, tobacco/tobacco 2, tobacco/tobacco 3, tobacco/eggplant 1, tobacco/eggplant 2, and tobacco/eggplant 3) were included in this study. Total RNA was isolated from the leaves using a Qiagen Plant RNA Mini Kit, as previously described [28]. All possible DNA contamination was removed by treating RNA with RNase-free DNase I (TaKaRa, Dalian, China) at 37 °C for 30 min. Gel electrophoresis was applied to check RNA quality. The concentration of RNA was determined by detecting absorbance at 260 nm using Agilent 2100 BioAnalyzer (Agilent Technologies, USA) and NanoDrop ND-2000 Spectrophotometer (Thermo Scientific, USA). mRNA was extracted from total RNA by utilizing magnetic oligo (dT) beads Then, based on the random hexamer-primed reverse transcription, the first-strand cDNA was synthetized. Next, using buffer, RNase H, DNA polymerase I, and dNTPs, second-strand cDNA was obtained. Short fragment was purified with a QIAquick Gel Extraction Kit (Qiagen, Frankfurt, Gremany) and resolved for adaptor ligation and end reparation. Approximately 200 bp cDNA fragments were isolated after gel electrophoresis. Finally, following the manufacturer’s instructions, the samples were performed single end read sequencing with 50 cycles (Illumina HiSeq 2000).

The sequencing data were deposited in National Center of Biotechnology Information database (https://www.ncbi.nlm.nih.gov/bioproject/PRJNA626466).

Quality control was first performed for the raw data of six samples, and the clean data were obtained. The clean reads were mapped to the reference genome of *N. tabacum* (assembly Ntab-TN90) (ftp://ftp.ncbi.nlm.nih.gov/genomes/all/GCF/000/715/135/GCF_000715135.1_Ntab-TN90/GCF_000715135.1_Ntab-TN90_genomic.fna.gz) using tophat software [29] (v2.1.0). The parameter was set to default. Read counts information for each gene were obtained based on tobacco genome annotation information (ftp://ftp.ncbi.nlm.nih.gov/genomes/all/GCF/000/715/135/GCF_000715135.1_Ntab-TN90/GCF_000715135.1_Ntab-TN90_genomic.gff.gz) using FeatureCounts software [30] (v1.6.0).

### Correlation analysis and PCA between samples

The expression level correlation between samples is an important index to test the reliability of the experiment and whether the sample selection is reasonable or not. We used cor function (https://stat.ethz.ch/R-manual/R-devel/library/stats/html/cor.html) in R3.4.1 to calculate the Pearson correlation coefficient (P). The closer the P to 1, the higher the similarity in the expression patterns between samples.

PCA aims to transform the multi-index into a few comprehensive indexes using dimension reduction. After PCA, the first two principal components were selected. According to the scores of the principal components, the distribution of the samples on the 2D plane could be drawn, and the classification of the samples could be determined from the graph. In this study, the prcomp function was used (https://stat.ethz.ch/R-manual/R-devel/library/stats/html/prcomp.html) to perform dimension reduction, and the ggfortify (Version: 0.4.5, https://mirrors.tuna.tsinghua.edu.cn/CRAN/bin/windows/contrib/3.4/ggfortify_0.4.5.zip) package was used to construct the PCA diagram.

### Screening of DEGs

Data normalization for raw counts was performed using the topological MM algorithm in the edgeR package [31, 32] (Version:3.4). Meanwhile, it was converted to the logCPM value, and DEGs analysis for the tobacco/eggplant group vs. the tobacco/tobacco group was conducted. The corresponding *p*-value and log-fold change (FC) for all genes were obtained, and *p*-value < 0.05 and |logFC| > 1 (|FC| > 2) were set as the threshold values.

### GO and KEGG pathway enrichment analysis

All obtained genes were mapped to the SWISS-PROT database [33], and GO terms, including molecular function (MF), cellular component (CC), and biological process (BP), for each gene were obtained. KEGG pathway analysis was conducted using the online automatic annotation web site KAAS [34] (https://www.genome.jp/tools/kaas/). The parameters were set as follows: search program = blast, organism = nta, and assignment method = BBH (bi-directional best hit).

Then, GO and KEGG pathway enrichment analyses for DEGs were conducted using R package GOstats (version: 2.40.0) [35]. Terms (GO-BP, GO-MF, GO CC, and KEGG pathway) with at least five genes were selected as the enrichment background. Finally, terms with *p*-value < 0.05 were regarded as significantly enriched results.

### PPI network construction and network degree analysis

STRING [36] (Version: 10.0, http://www.string-db.org/) database was used to predict interactions between proteins encoded by genes. There were no interactions between *N. tabacum*, which was an allotetraploid produced by the interspecific hybridization between *N. sylvestris* and *N. tomentosiformis* [37]. Therefore, DEGs were mapped to the protein sequence of *N. sylvestris* and *N. tomentosiformis* to obtain the corresponding relationship between DEGs and *N. sylvestris* as well as *N. tomentosiformis*, respectively. Then, the PPI network for *N. tabacum* was constructed by searching the interactions of *N. sylvestris* and *N. tomentosiformis* in the database. The PPI score was set as 0.4 (medium confidence). After obtaining the PPI pairs, the network was constructed using Cytoscape software (version 3.4.0, http://chianti.ucsd.edu/cytoscape-3.4.0/) [38].

The network degree analysis for the node was conducted using the CytoNCA plugin [39] (Version 2.1.6, http://apps.cytoscape.org/apps/cytonca). The parameter was set as without weight. The important nodes involved in protein interactions in the PPI network (hub protein) were obtained by ranking of the degree of each node.

### PPI module analysis

The modules with score > 5 were screened using the MCODE plugin [40] in Cytoscape software, and the parameter was set to default (Degree Cutoff = 2, Node Score Cutoff = 0.2, K-core = 2, and Max. Depth = 100). GO and KEGG pathway enrichment analyses were conducted using the R package GOstats. Terms (GO-BP, GO-MF, GO-CC, and KEGG pathway) with at least five genes were selected as the enrichment background. Finally, terms with *p*-value < 0.05 were regarded as significantly enriched results.

### TFs prediction and TF-target network construction

The online database PlantRegMap [41, 42] (http://plantregmap.cbi.pku.edu.cn/) was used to predict the TFs for DEGs, and the differentially expressed TFs and corresponding differentially expressed target genes. The parameters were set as follows: Species = *N. tabacum*, Organ = All, Method = Motif, Mode = Genes (retrieve regulations among them). After obtaining the TF target, the TF target network was constructed using Cytoscape software.

## Supplementary information

**Additional file 1: Fig. S1.** The feeding selection behavior of *Heliothis assulta* on tobacco leaves after grafting with eggplant.

**Additional file 2: Fig. S2.a:** Correlation heatmaps between two samples based on expression abundance; **b:** principal component analysis (PCA) between two samples; yanyan = tobacco/tobacco; yanqie = tobacco/eggplant.

**Additional file 3: Fig. S3.** The heatmap (**a**) and volcano plot (**b**) for differentially expressed genes (DEGs).

**Additional file 4: Fig. S4.** The top 10 results of gene ontology (GO) and Kyoto Encyclopedia of Genes and Genomes (KEGG) pathway enrichment analyses for differentially expressed genes (DEGs).

**Additional file 5: Fig. S5.** Protein-protein interaction (PPI) networks for *Nicotiana sylvestris* (a) and *Nicotiana tomentosiformis* (b). Red: upregulated proteins; green: downregulated proteins; deeper color indicates bigger |logFC|; gray lines: interactions between proteins; bigger nodes indicate bigger degrees.

**Additional file 6: Fig. S6.** a: A module identified in the protein-protein interaction (PPI) network for *Nicotiana sylvestris*; **b:** the top 10 results of gene ontology (GO) and Kyoto Encyclopedia of Genes and Genomes (KEGG) pathway enrichment analyses for the 13 genes.

**Additional file 7: Fig. S7.** Transcription factors (TFs) target regulating network for differentially expressed genes (DEGs). Red square: upregulated target genes; green square: downregulated target genes; light-purple inverted triangle: downregulated TFs; yellow triangle: upregulated TFs; black arrow line: TFs regulating target genes.

**Additional file 8: Fig. S8.** a: The arrangement of fresh tobacco leaves before *Heliothis assulta* consumption; **b:** the condition of the leaves after 12 h.

**Additional file 9: Table S1.** Effect of tobacco and eggplant grafting on amino acids in fresh tobacco. **Table S2.** Effect of tobacco and eggplant grafting on amino acids in cured tobacco. **Table S3.** Effect of tobacco and eggplant grafting on the content of TSNAs in flue-cured tobacco.

## Data Availability

The sequencing data were deposited in National Center of Biotechnology Information database (https://www.ncbi.nlm.nih.gov/bioproject/PRJNA626466). The datasets used and/or analyzed during the current study are available from the corresponding author on reasonable request.

## Abbreviations

PCA: Principal component analysis
DEGs: Differentially expressed genes
GO: Gene ontolog
KEGG: Kyoto Encyclopedia of Genes and Genomes
PPI: Protein-protein interaction
TF: Transcription factor
TSNA: Tobacco-specific nitrosamine
MF: Molecular function
CC: Cellular component
BP: Biological process
FC: Fold change
NNN: 4-(methylnitrosamino)-1-(3-pyridyl)-1-butanone
NAT: (R,S)-N-nitrosoanatabine
NAB: (R,S)-N-nitrosoanabasine
NNK: 4-(methylnitrosamino)-1-(3-pyridyl)-1-butanone
