# Supplementary figures and images for: Reducing the nicotine content of tobacco by grafting with eggplant
Source: BMC Plant Biol. 2020 Jun 22;20:285. doi: 10.1186/s12870-020-02459-4 (PMC7310140; doi:10.1186/s12870-020-02459-4)

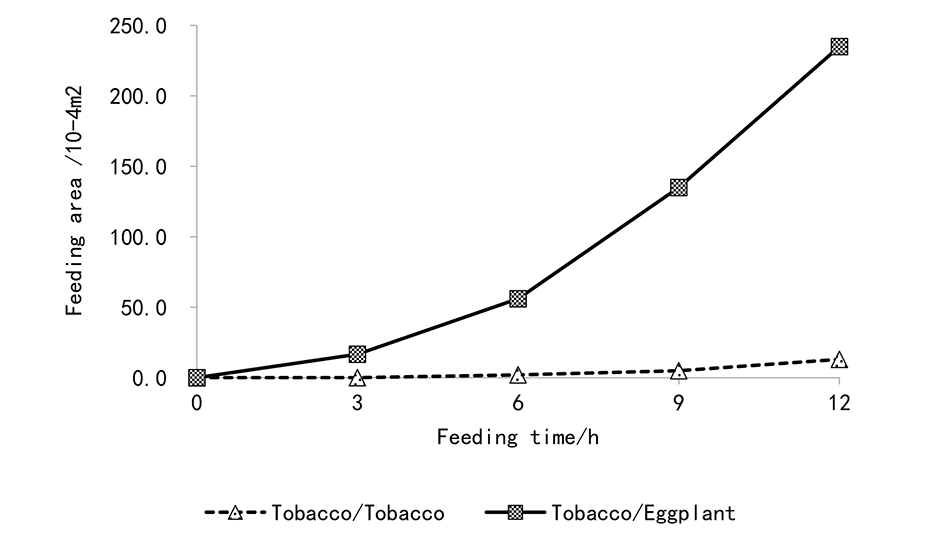

Supplement: Supplementary file 1 — Additional file 1: Fig. S1. The feeding selection behavior of Heliothis assulta on tobacco leaves after grafting with eggplant. [file 12870_2020_2459_MOESM1_ESM.tif]

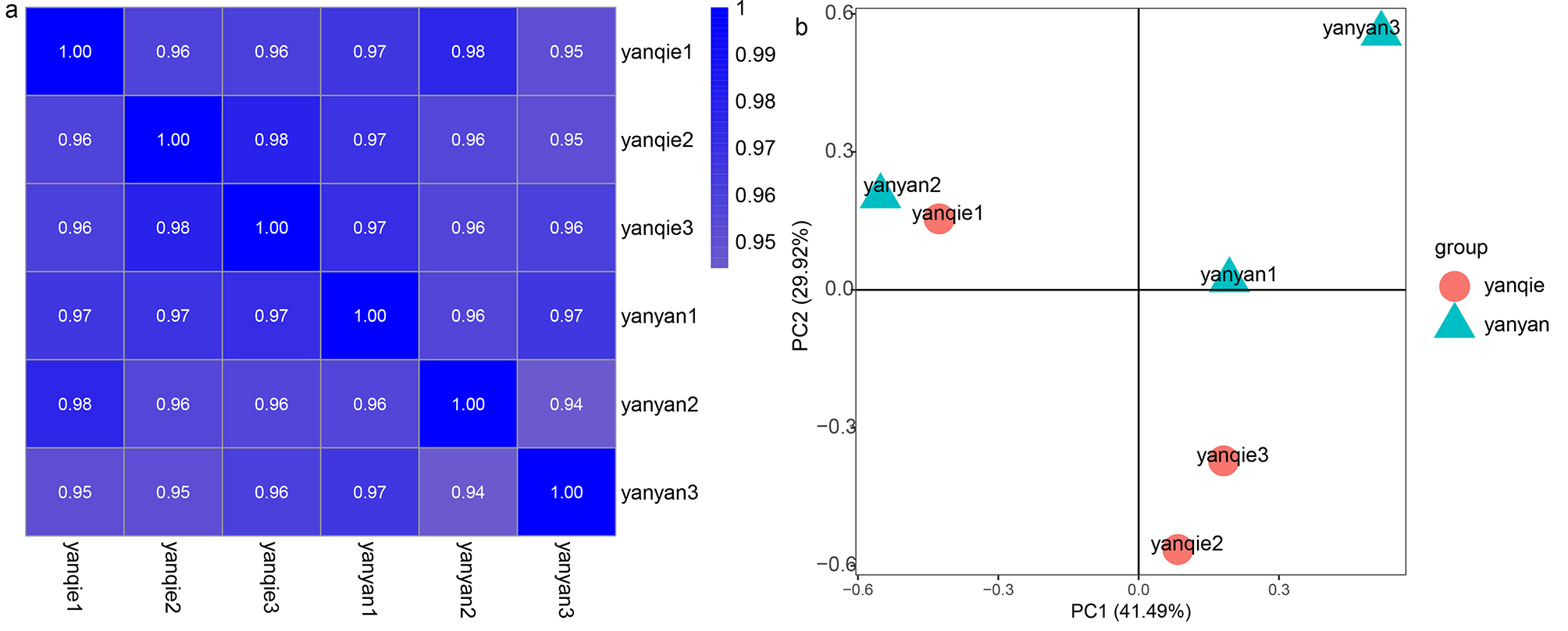

Supplement: Supplementary file 2 — Additional file 2: Fig. S2.a: Correlation heatmaps between two samples based on expression abundance; b: principal component analysis (PCA) between two samples; yanyan = tobacco/tobacco; yanqie = tobacco/eggplant. [file 12870_2020_2459_MOESM2_ESM.tif]

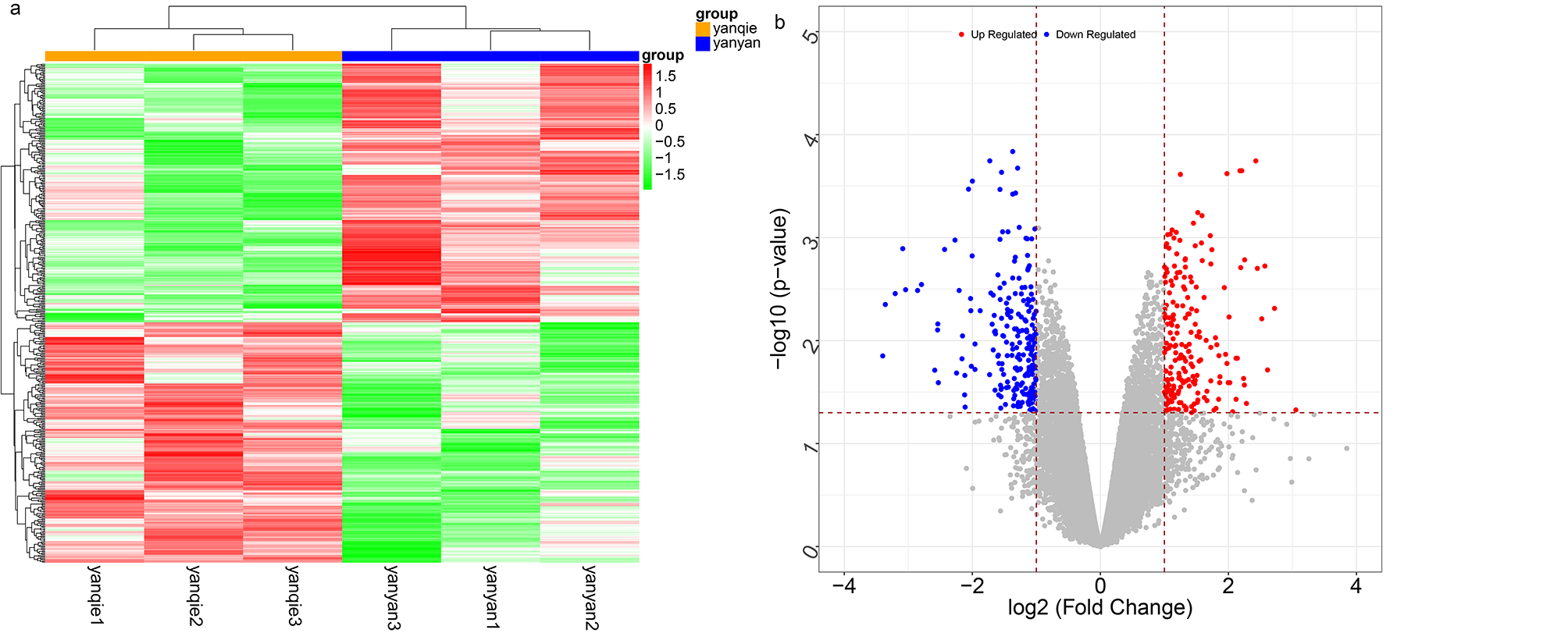

Supplement: Supplementary file 3 — Additional file 3: Fig. S3. The heatmap (a) and volcano plot (b) for differentially expressed genes (DEGs). [file 12870_2020_2459_MOESM3_ESM.tif]

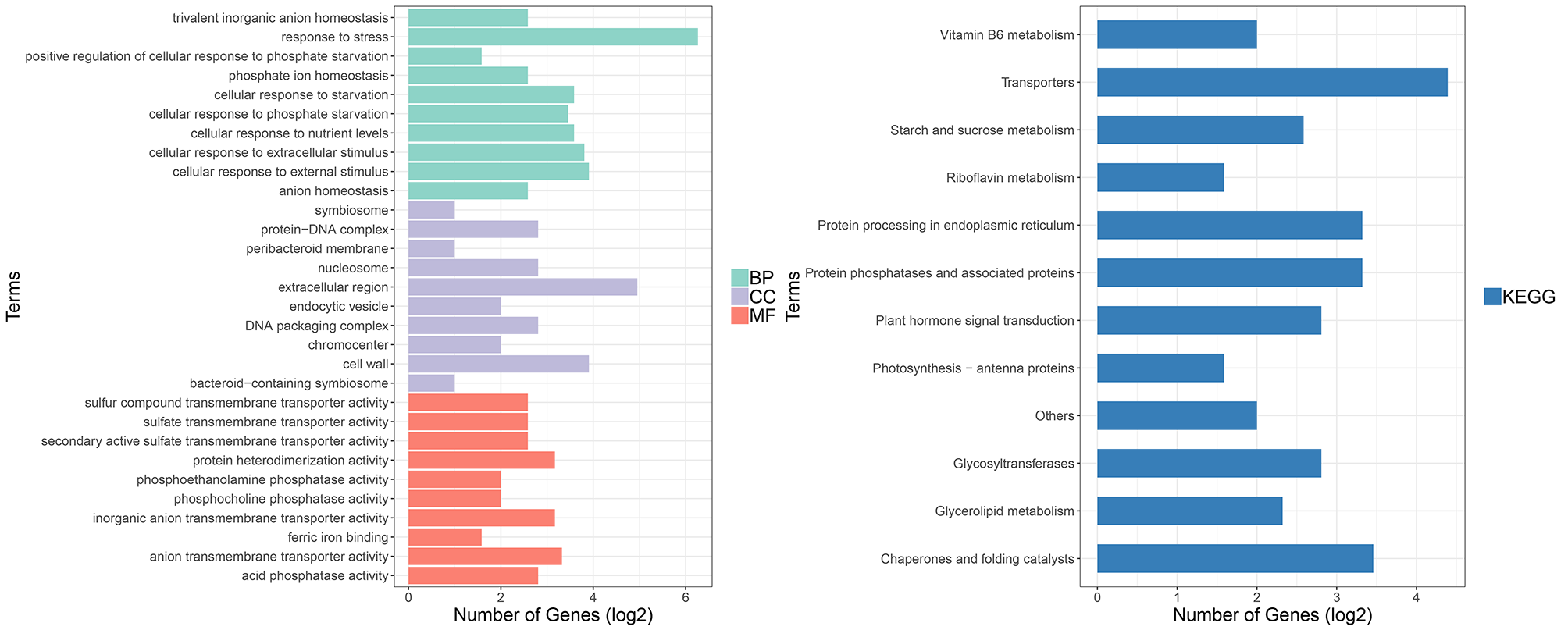

Supplement: Supplementary file 4 — Additional file 4: Fig. S4. The top 10 results of gene ontology (GO) and Kyoto Encyclopedia of Genes and Genomes (KEGG) pathway enrichment analyses for differentially expressed genes (DEGs). [file 12870_2020_2459_MOESM4_ESM.tif]

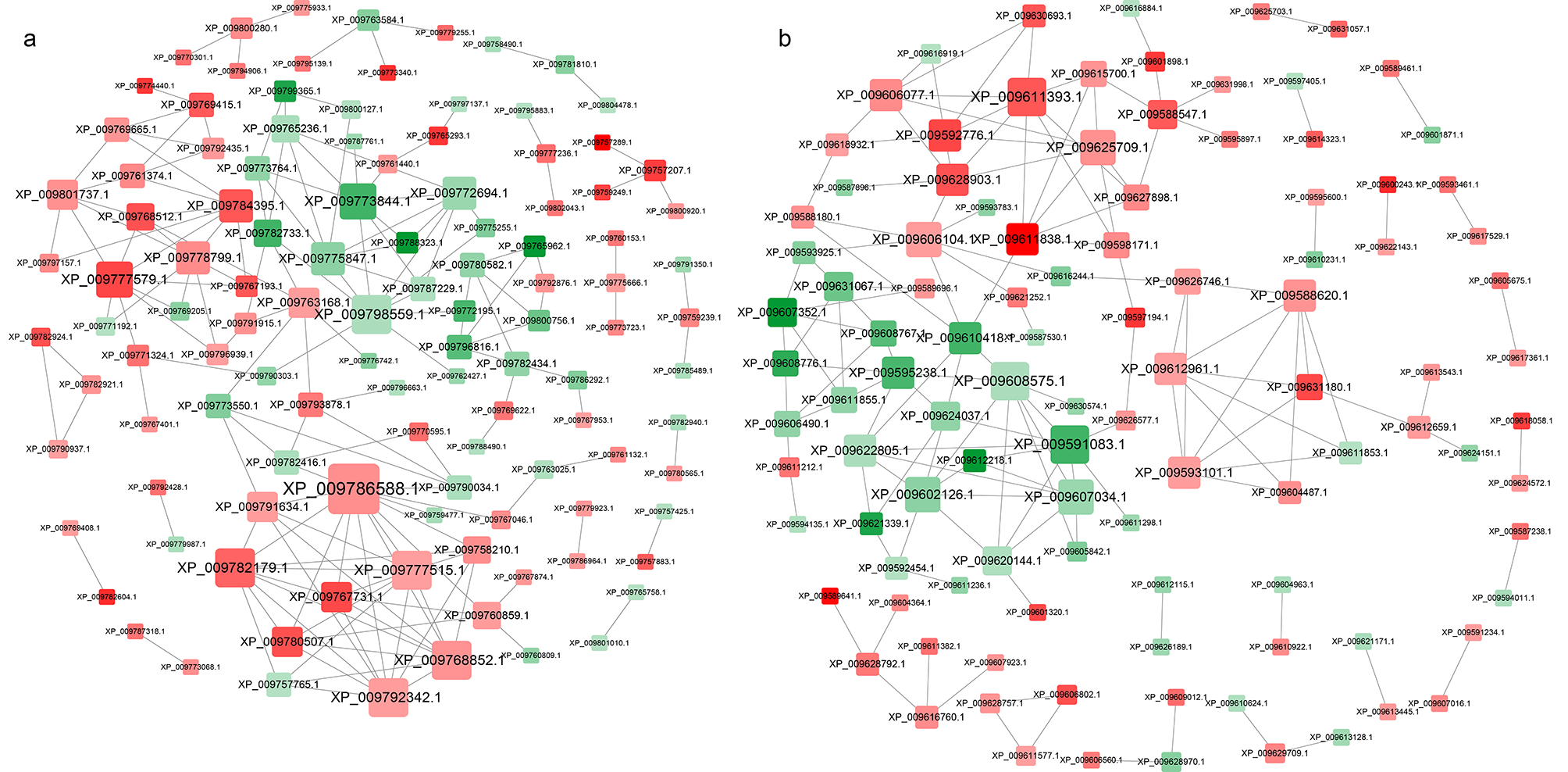

Supplement: Supplementary file 5 — Additional file 5: Fig. S5. Protein-protein interaction (PPI) networks for Nicotiana sylvestris (a) and Nicotiana tomentosiformis (b). Red: upregulated proteins; green: downregulated proteins; deeper color indicates bigger |logFC|; gray lines: interactions between proteins; bigger nodes indicate bigger degrees. [file 12870_2020_2459_MOESM5_ESM.tif]

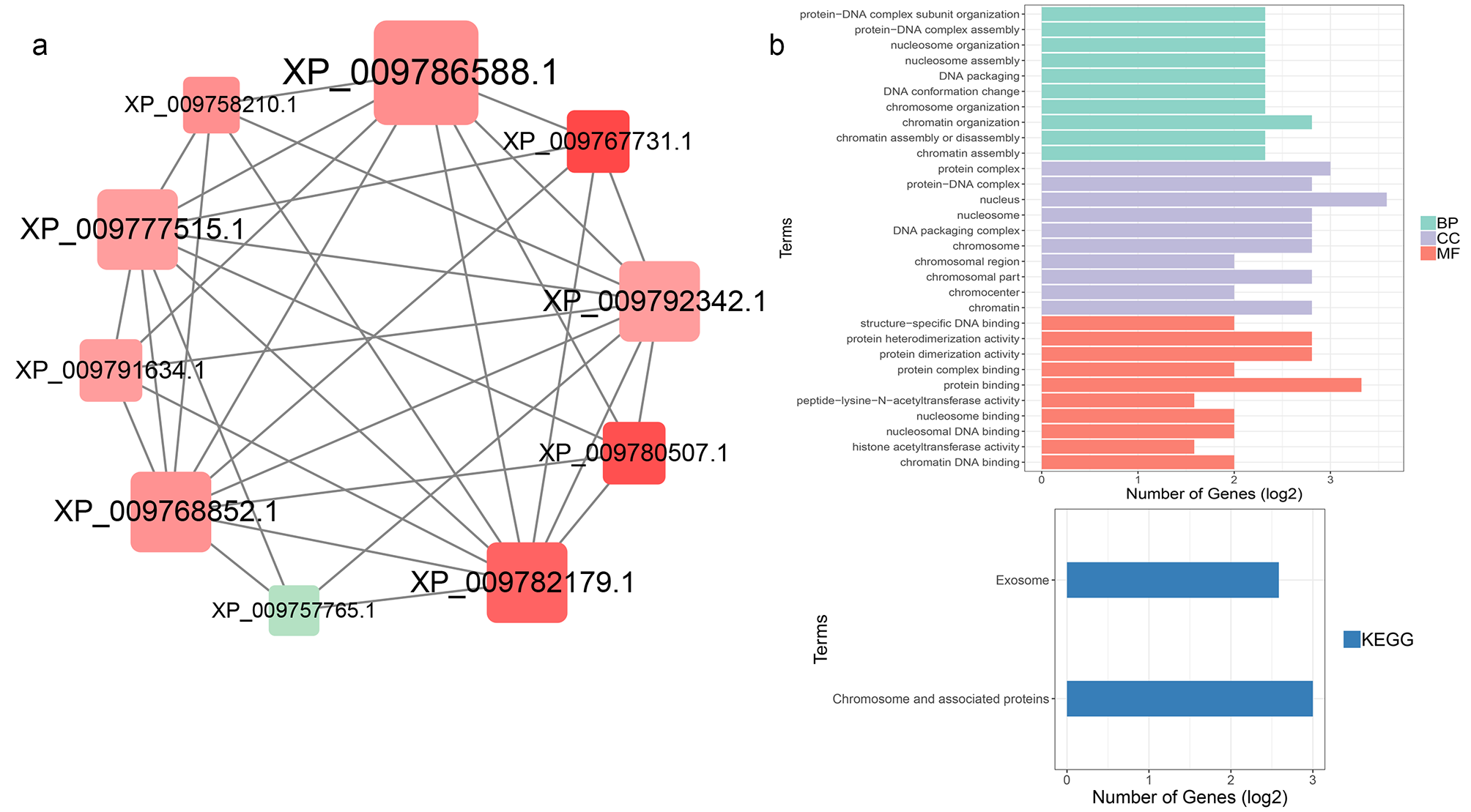

Supplement: Supplementary file 6 — Additional file 6: Fig. S6. a: A module identified in the protein-protein interaction (PPI) network for Nicotiana sylvestris; b: the top 10 results of gene ontology (GO) and Kyoto Encyclopedia of Genes and Genomes (KEGG) pathway enrichment analyses for the 13 genes. [file 12870_2020_2459_MOESM6_ESM.tif]

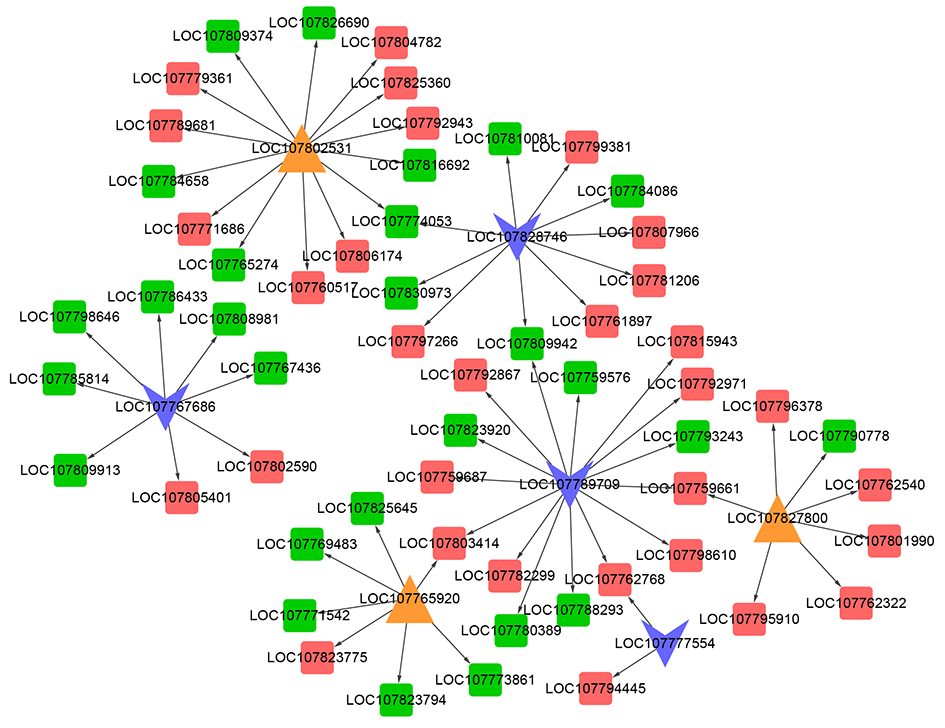

Supplement: Supplementary file 7 — Additional file 7: Fig. S7. Transcription factors (TFs) target regulating network for differentially expressed genes (DEGs). Red square: upregulated target genes; green square: downregulated target genes; light-purple inverted triangle: downregulated TFs; yellow triangle: upregulated TFs; black arrow line: TFs regulating target genes. [file 12870_2020_2459_MOESM7_ESM.tif]

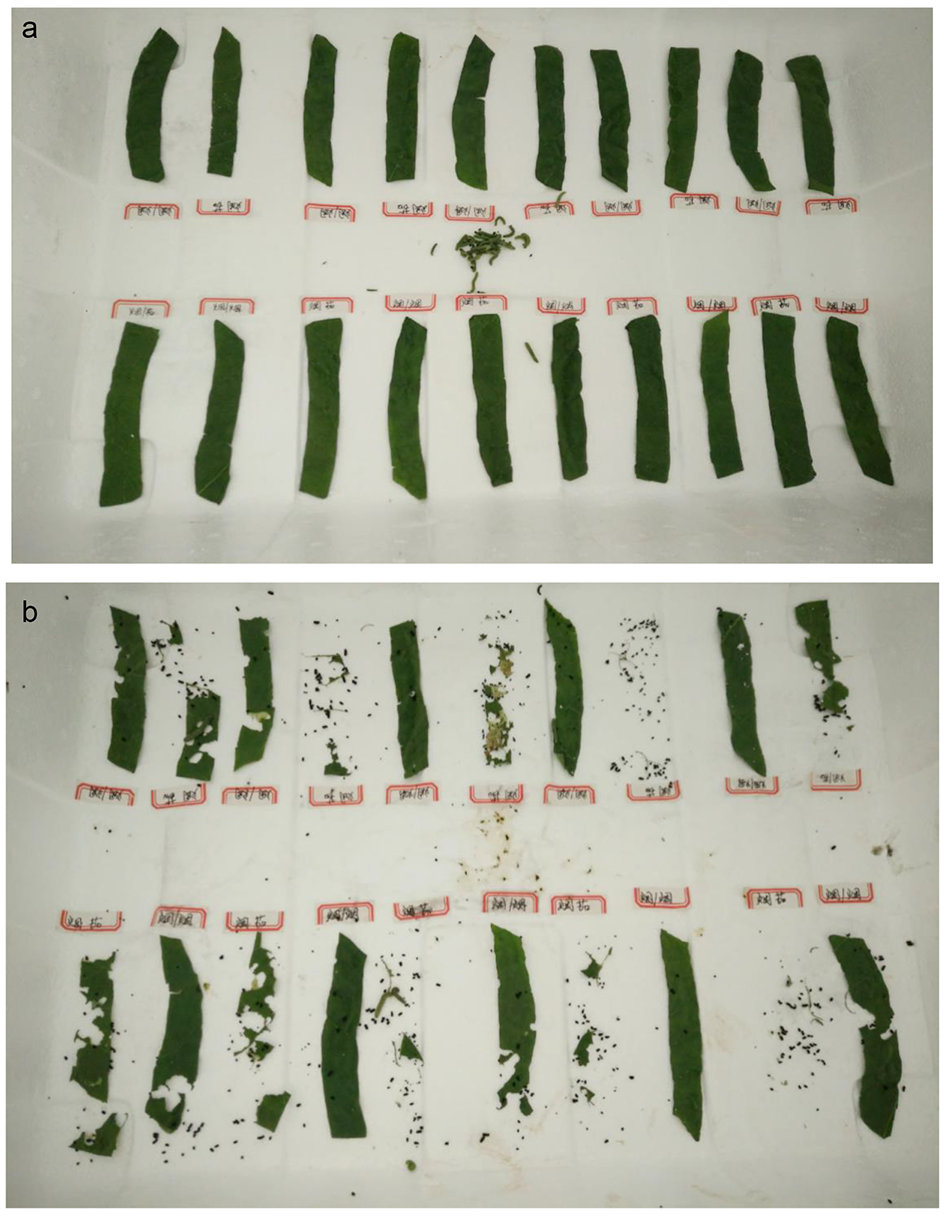

Supplement: Supplementary file 8 — Additional file 8: Fig. S8. a: The arrangement of fresh tobacco leaves before Heliothis assulta consumption; b: the condition of the leaves after 12 h. [file 12870_2020_2459_MOESM8_ESM.tif]
