# Supplementary material for: Reducing the nicotine content of tobacco by grafting with eggplant
Source: BMC Plant Biol. 2020 Jun 22;20:285. doi: 10.1186/s12870-020-02459-4 (PMC7310140; doi:10.1186/s12870-020-02459-4)
Supplement: Supplementary file 9 — Additional file 9: Table S1. Effect of tobacco and eggplant grafting on amino acids in fresh tobacco. Table S2. Effect of tobacco and eggplant grafting on amino acids in cured tobacco. Table S3. Effect of tobacco and eggplant grafting on the content of TSNAs in flue-cured tobacco. [file 12870_2020_2459_MOESM9_ESM.docx]

**Table S1.** Effect of tobacco and eggplant grafting on amino acids in fresh tobacco

| Treatments | Tobacco/tobacco(mg/g) | Tobacco/eggplant without hilling up(mg/g) | | Tobacco/eggplant without hilling up (mg/g) | |
| --- | --- | --- | --- | --- | --- |
|  |  | contents(mg/g) | Increase or decrease over the control% | contents(mg/g) | Increase or decrease over the control% |
| Aspartic acid (Asp) | 12.50±0.10c | 14.40±0.20b | 15.20 | 15.00±0.06a | 20.00 |
| Threonine (Thr) | 6.10±0.10c | 6.90±0.10b | 13.10 | 7.10±0.15a | 16.40 |
| Serine (Ser) | 5.80±0.20b | 6.40±0.10a | 10.30 | 6.60±0.06a | 13.80 |
| Glutamic acid (Glu) | 16.40±0.06c | 18.70±0.30b | 14.00 | 19.60±0.10a | 19.50 |
| Glycine (Gly) | 7.00±0.06b | 8.00±0.15a | 14.30 | 8.00±0.06a | 14.30 |
| Alanine (Ala) | 8.60±0.20b | 9.60±0.15a | 11.60 | 9.60±0.10a | 11.60 |
| Valine (Val) | 8.60±0.06b | 9.50±0.10a | 10.50 | 9.60±0.06a | 11.60 |
| Methionine (Met) | 1.90±0.10b | 2.20±0.15a | 15.80 | 2.20±0.06a | 15.80 |
| Isoleucine (Ile) | 5.80±0.06b | 6.60±0.20a | 13.80 | 6.60±0.10a | 13.80 |
| Leucine (Leu) | 11.40±0.10c | 12.80±0.25b | 12.30 | 13.10±0.15a | 14.90 |
| Tyrosine (Tyr) | 3.40±0.20a | 3.80±0.35a | 11.80 | 3.80±0.15a | 11.80 |
| Phenylalanine (Phe) | 9.30±0.10b | 10.10±0.10a | 8.60 | 10.30±0.10a | 10.80 |
| Lysine (Lys) | 9.20±0.15c | 10.00±0.06b | 8.70 | 10.20±0.06a | 10.90 |
| Histidine (His) | 3.20±0.20b | 3.40±0.06ab | 6.30 | 3.70±0.15a | 15.60 |
| Arginine (Arg) | 6.80±0.15b | 7.60±0.10a | 11.80 | 7.70±0.10a | 13.20 |
| Proline (Pro) | 9.80±0.10c | 11.90±0.20a | 21.40 | 11.00±0.20b | 12.20 |
| Total contents | 125.80±0.30b | 141.90±2.65a | 12.80 | 144.10±2.77a | 14.50 |

**Note:** The difference between treatments is significant (P < 0.05) if the same number is not marked with the same lowercase letter.

**Table S2.** Effect of tobacco and eggplant grafting on amino acids in cured tobacco

| Treatments | Tobacco/tobacco (mg/g) | Tobacco/eggplant without hilling up (mg/g) | | Tobacco/eggplant without hilling up (mg/g) | |
| --- | --- | --- | --- | --- | --- |
|  |  | Contents (mg/g) | Increase or decrease over the control % | Contents (mg/g) | Increase or decrease over the control % |
| Aspartic acid (Asp) | 18.00±0.06c | 21.60±0.15b | 20.00 | 22.20±0.10a | 23.33 |
| Threonine (Thr) | 3.40±0.10b | 4.40±0.06a | 29.41 | 4.20±0.06a | 23.53 |
| Serine (Ser) | 3.40±0.06c | 4.60±0.06a | 35.29 | 4.20±0.06b | 23.53 |
| Glutamic acid (Glu) | 22.40±0.06b | 19.90±0.10c | 11.16 | 26.20±0.10a | 16.96 |
| Glycine (Gly) | 3.80±0.10c | 5.20±0.10a | 36.84 | 4.800.20±b | 26.32 |
| Alanine (Ala) | 5.40±0.21c | 6.10±0.10b | 12.96 | 6.60±0.15a | 22.22 |
| Valine (Val) | 4.60±0.10b | 5.30±0.15a | 15.22 | 5.60±0.10a | 21.74 |
| Methionine (Met) | 1.40±0.10b | 1.70±0.10a | 21.43 | 1.60±0.06ab | 14.29 |
| Isoleucine (Ile) | 2.80±0.15b | 3.80±0.10a | 35.71 | 3.90±0.10a | 39.29 |
| Leucine (Leu) | 5.40±0.10c | 6.50±0.10b | 20.37 | 6.90±0.10a | 27.78 |
| Tyrosine (Tyr) | 1.80±0.20ab | 1.60±0.06b | 11.11 | 2.20±0.25a | 22.22 |
| Phenylalanine (Phe) | 8.20±0.06b | 7.40±0.06c | 9.76 | 9.00±0.10a | 9.76 |
| Lysine (Lys) | 6.00±0.15c | 7.40±0.06b | 23.33 | 8.00±0.10a | 33.33 |
| Histidine (His) | 3.60±0.10b | 3.50±0.10b | 2.78 | 4.20±0.06a | 16.67 |
| Arginine (Arg) | 3.80±0.10c | 4.50±0.10b | 18.42 | 4.80±0.10a | 26.32 |
| Proline (Pro) | 12.90±0.10c | 25.10±0.10a | 94.57 | 15.40±0.25b | 19.38 |
| Total contents | 106.90±1.06b | 128.60±2.25a | 20.30 | 129.80±1.73a | 21.42 |

**Note:** The difference between treatments is significant (P < 0.05) if the same number is not marked with the same lowercase letter.

**Table S3.** Effect of tobacco and eggplant grafting on the content of TSNAs in flue-cured tobacco

| Position | Treatments | NNN(ng/g) | NAT(ng/g) | NAB(ng/g) | NNK(ng/g) | Total(ng/g) |
| --- | --- | --- | --- | --- | --- | --- |
| Upper leaf | Tobacco/tobacco | 49.40±0.30aA | 22.12±0.60aA | 3.76±0.04cC | 18.44±0.18aA | 93.72±0.72aA |
|  | Tobacco / eggplant without hilling up | 8.54±0.01cC | 12.53±0.27cC | 4.41±0.09bB | 3.81±0.09cC | 29.29±0.77cC |
|  | Tobacco / eggplant with hilling up | 15.37±0.48bB | 17.73±0.52bB | 6.02±0.01aA | 12.39±0.23bB | 51.51±1.49bB |
| Middle leaf | Tobacco/tobacco | 52.50±0.32aA | 25.91±0.82aA | 2.24±0.03cC | 19.90±0.41aA | 100.56±1.19aA |
|  | Tobacco / eggplant without hilling up | 10.67±0.08cC | 14.22±0.35cC | 2.63±0.07bB | 4.02±0.07cC | 31.53±0.80cC |
|  | Tobacco / eggplant with hilling up | 18.57±0.05bB | 19.21±0.02bB | 3.59±0.10aA | 13.37±0.25bB | 54.74±0.22bB |

**Note:** The difference between treatments is significant (P < 0.05) if the same number is not marked with the same lowercase letter and the difference between the treatments is extremely significant (P < 0.01) without the same capital letter.
